# Loss of chromosome Y in blood, but not in brain, of suicide completers

**DOI:** 10.1371/journal.pone.0190667

**Published:** 2018-01-04

**Authors:** Atsushi Kimura, Akitoyo Hishimoto, Ikuo Otsuka, Satoshi Okazaki, Shuken Boku, Tadasu Horai, Takeshi Izumi, Motonori Takahashi, Yasuhiro Ueno, Osamu Shirakawa, Ichiro Sora

**Affiliations:** 1 Department of Psychiatry, Kobe University Graduate School of Medicine, Kobe, Japan; 2 Department of Neuropharmacology, Hokkaido University Graduate School of Medicine, Sapporo, Japan; 3 Division of Legal Medicine, Department of Community Medicine and Social Health Science, Kobe University Graduate School of Medicine, Kobe, Japan; 4 Department of Neuropsychiatry, Kindai University Faculty of Medicine, Osaka, Japan; Hudson Institute, AUSTRALIA

## Abstract

Men have a higher rate of completed suicide than women, which suggests that sex chromosome abnormalities may be related to the pathophysiology of suicide. Recent studies have found an aberrant loss of chromosome Y (LOY) in various diseases; however, no study has investigated whether there is an association between LOY and suicide. The purpose of this study was to determine whether LOY occurs in men who completed suicide. Our study consisted of 286 male Japanese subjects comprised of 140 suicide completers without severe physical illness (130 post-mortem samples of peripheral blood and 10 brains) and 146 age-matched control subjects (130 peripheral blood samples from healthy individuals and 16 post-mortem brains). LOY was measured as the chromosome Y/chromosome X ratio of the fluorescent signal of co-amplified short sequences from the Y-X homologous amelogenin genes (*AMELY* and *AMELX*). Regression analyses showed that LOY in the blood of suicide completers was significantly more frequent than that found in controls (odds ratio = 3.50, 95% confidence interval = 1.21–10.10), but not in the dorsolateral prefrontal cortex (DLPFC) region of brain. Normal age-dependent LOY in blood was found in healthy controls (r = -0.353, p < 0.001), which was not seen in suicide completers (r = -0.119, p = 0.177). DLPFC tissue had age-dependent LOY (B = -0.002, p = 0.015), which was independent of phenotype. To our knowledge, this is the first study demonstrating that LOY in blood is associated with suicide completion. In addition, our findings are the first to also indicate that age-dependent LOY may occur not only in blood, but also in specific brain regions.

## Introduction

Suicide is a significant public health problem worldwide with approximately 1 million suicides each year. Emerging evidence from genetic studies suggests an underlying genetic pathophysiology in suicidal behavior in people with severe stress or mental disorders [1,2]. In most countries, men have a much higher rate of completed suicide compared to women as well as greater lethality of the suicide method [3–5], although the pathophysiology of this sex difference remains unclear. Findings from numerous studies have indicated that genetic factors, and in particular the sex chromosomes, contribute to abnormal psychiatric conditions and to the sex differences reported for various psychological problems [6]. For instance, Klinefelter syndrome (one or more supernumerary X chromosomes in men) has been associated with violent behavior [7,8], also known as the factor related to suicide lethality [9].

The Y chromosome is recognized for its role in sex determination and normal sperm production, but it was considered a genetic wasteland and its characterization lagged behind the rest of the genome [10]. However, recent studies have shown that the human Y chromosome contains many genes [10,11], and interestingly, there is a mosaic loss of chromosome Y (LOY) in peripheral blood cells of aging men. LOY can be detected in ≥ 10% of normal blood cells in at least 15% of aging men ≥ 70 years of age, and may at least partly explain why men on average have shorter lifespans than women [12–14]. A recent genome-wide association study (GWAS) using two independent cohorts (ULSAM and PIVUS) showed that the median survival time among men with LOY was 5.5 years shorter and approximately half as long compared to those without LOY [12]. Other studies identified that smoking and several single nucleotide polymorphisms (SNPs) were associated with LOY [13, 15,16]. In addition to proposing that LOY in peripheral blood may be a male-specific risk factor in Alzheimer’s disease, Dumanski et al. [17] hypothesized that LOY in blood cells may indicate chromosomal instability in other cell types such as neurons, although no previous study has investigated whether LOY occurs in brain tissue.

In this study, we hypothesized that the male-specific chromosomal event LOY contributes towards the sex difference found in completed suicide. Furthermore, because most psychiatric problems including suicide may be derived from a wide range of brain abnormalities, we ascertained the presence of LOY not only in post-mortem blood, but also brain from male suicide completers and male controls.

## Materials and methods

### Subjects

Our study design and all related procedures were performed in accordance with the Declaration of Helsinki. This study was approved by the Ethical Committee for Genetic Studies of the Kobe University Graduate School of Medicine. Subject selection and sample preparation were performed as previously described [2].

All subjects were of Japanese descent. Autopsies on suicide completers were conducted at the Division of Legal Medicine in the Department of Community Medicine and Social Health Science at the Kobe University Graduate School of Medicine. The determination of a “completed suicide” was made through discussions with the Medical Examiner's Office of Hyogo Prefecture and the Division of Legal Medicine in the Kobe University Graduate School of Medicine. Written informed consent was obtained from all participants and from the families of deceased subjects from which post-mortem blood and brain samples were obtained. Individuals with known severe physical illnesses (cardiovascular diseases, cerebral infarction, diabetes, bone marrow diseases, and cancer) were excluded from the study.

Post-mortem peripheral blood samples were obtained from male suicide completers (n = 130), and from age- and sex-matched healthy living controls (n = 130). Healthy volunteers were recruited from the main islands of Japan and included medical students, hospital workers, and individuals from the general population. No control subjects were related to each other or manifested psychiatric problems in unstructured interviews independently conducted by two psychiatrists using Diagnostic and Statistical Manual of Mental Disorders 4^th^ edition (DSM-IV) criteria. During the interview, all control subjects were checked for a personal and family history of psychiatric disorders based on DSM-IV and/or suicidal behaviors. We excluded control subjects with a personal or family history of psychiatric disorders or suicidal behaviors. We obtained information regarding smoking status in most of suicide completers (n: never smokers = 89, former smokers = 5, current smokers = 32, unknown individuals = 4), although those information was not available for control individuals. Autopsied brains were obtained from 10 suicide victims and 16 control subjects. The dorsolateral prefrontal cortex (DLPFC) was dissected on dry ice for subsequent DNA extraction. Demographic and clinical data of our cohorts are shown in Table 1.

**Table 1:** Demographic and clinical details of male subjects.

	Peripheral blood			Prefrontal cortex		
	Suicide (n = 130)	Control (n = 130)	p^a^	Suicide (n = 10)	Control (n = 16)	p^a^
Average age in years (± s.d.)	46.2 (14.0)	45.4 (13.4)	0.526	51.7 (17.5)	55.9 (18.3)	0.496
PMI in hours (± s.d.)	20.3 (10.5)			15.1 (7.1)	17.7 (11.2)	0.777
Suicide method						
Hanging	92			1		
Jumping from a height	19			3		
Gas suffocation	16			1		
Jumping in front of a vehicle	1					
Self-inflicted penetrating wounds	1			2		
Drowning	1			1		
Overdosing				2		
Comorbid psychiatric disease						
Depressive disorders	28			2		
Bipolar disorders	4					
Schizophrenia	13			1		
Anxiety disorders	6					
Personality disorders	2					
Alcohol-related disorders	2					
Mental disorders	1					
Unknown	7			3		

Abbreviations: s.d., standard deviation; PMI, post-mortem interval.

^a^ p-values were calculated using Mann-Whitney tests.

### Assessment of LOY in blood and brain samples

Blood and brain samples were stored at -80°C before use. DNA was extracted using either the QIAamp DNA Blood Midi Kit or the DNeasy Blood & Tissue Kit (Qiagen Inc., Valencia, CA, USA) as appropriate. Extracted DNA was quantified and underwent quality control using a NanoDrop spectrophotometer (Thermo Scientific, Wilmington, DE, USA). LOY was determined using quantitative fluorescence polymerase chain reaction as previously described [18–20]. Briefly, the relative amount of Y chromosome was determined as a chromosome Y/chromosome X ratio (Y/X ratio) of fluorescent signal of co-amplified short sequences from the Y-X homologous amelogenin genes (*AMELY* and *AMELX*) discriminated by a 6-base pair (bp) deletion in intron 1 in *AMELX* that is not found in *AMELY*. Deletions or duplications in AMELY region were excluded by a co-amplified homologous sequence primer set for *MYPT2* on chromosome 1 and chromosome Y. In addition, using another co-amplified homologous sequence set for *TAF9B* found on chromosome 3 and the X chromosome, we confirmed no false positive cases of X chromosome loss or gain in this study. The primer sequences and cycling conditions are described in S1 Table. PCR products were run on an Applied Biosystems 3510 XL Genetic Analyzer with LIZ500 as an internal standard and analyzed with GeneMapper 4.1 (Life Technologies, Carlsbad, CA, USA). Because co-amplified fragments from the X and Y chromosomes have a 6 bp difference (106 bp versus 112 bp, respectively), they are readily separated and quantified by capillary electrophoresis. Laboratory personnel were blinded regarding case-control status and the sample order was randomized in each batch.

### SNP selection and genotyping

From LOY-associated SNPs previously identified by European GWASs (all p < 5 × 10^−8^) [15,16], we selected and genotyped five SNPs (rs13191948, rs4721217, rs2887399, rs12448368, and rs11082396) with the following criteria; a minor allele frequency > 0.01 in the Japanese population based on the 1000 Genomes Project Phase 3 (http://phase3browser.1000genomes.org/index.html), available TaqMan SNP genotyping probes from the Applied Biosystems database (http://bioinfo.appliedbiosystems.com/genome-database/), and genotype call rate > 0.98. Genotyping was performed on a 7500 Real-Time PCR System (Applied Biosystems, Foster City, CA, USA) according to the manufacturer’s protocol.

### Statistical analysis

Statistical analysis was performed using R Version 3.2.2 (https://www.r-project.org). Mann-Whitney tests were performed to analyze between-group comparisons of continuous variables. To account for the non-normal distribution of Y/X ratios in our blood samples (Shapiro-Wilk test; p < 0.001) and the need for controlling age effect, we performed binary and multiple logistic regression analyses coding Y/X ratios < 0.9 as LOY and Y/X ratios ≥ 0.9 as normal. This threshold was based on the previous findings; Detection of LOY from SNP-array data is robust and reproducible when LOY occurs in ≥10% of the nucleated cells in a blood sample [13,14,17]. Because the Y/X ratios in brain tissue samples from the DLPFC were normally distributed, we performed multiple linear regression analysis of the Y/X ratio in the DLPFC of suicide completers and controls. Regression analyses were used with covariates [age, postmortem interval (PMI) and SNP genotype] as appropriate. Dummy variables were used as necessary (phenotype, control = 0 and suicide = 1; SNP genotype, major-major = 0, major-minor = 1 and minor-minor = 2; smoking status [multiple comparisons], never/former smokers = 0 vs. current smokers = 1, never smokers = 0 vs. current/former smokers = 1, never smokers = 0 vs. former smokers = 1 vs. current smokers = 2). Spearman correlation coefficients were performed to assess the relationships between LOY and age. Statistical significance was defined as two-tailed p-values < 0.05. Further, we used Haploview version 4.2 (http://www.broadinstitute.org/haploview/haploview) [21] to determine Hardy-Weinberg equilibrium and allele frequencies for each SNP. Allele and genotype association analyses between LOY and control groups were performed by the χ^2^ test or the Cochran-Armitage trend test as appropriate, using Haploview. Power analysis was performed using PS v2.1.3.1 [22]. Statistical significance for the SNP association study was defined as a two-tailed p-value < 0.05. Permutation tests based on 10,000 replications were performed to calculate the corrected p values of the allelic.

## Results

### LOY analysis in peripheral blood

**Fig 1:**
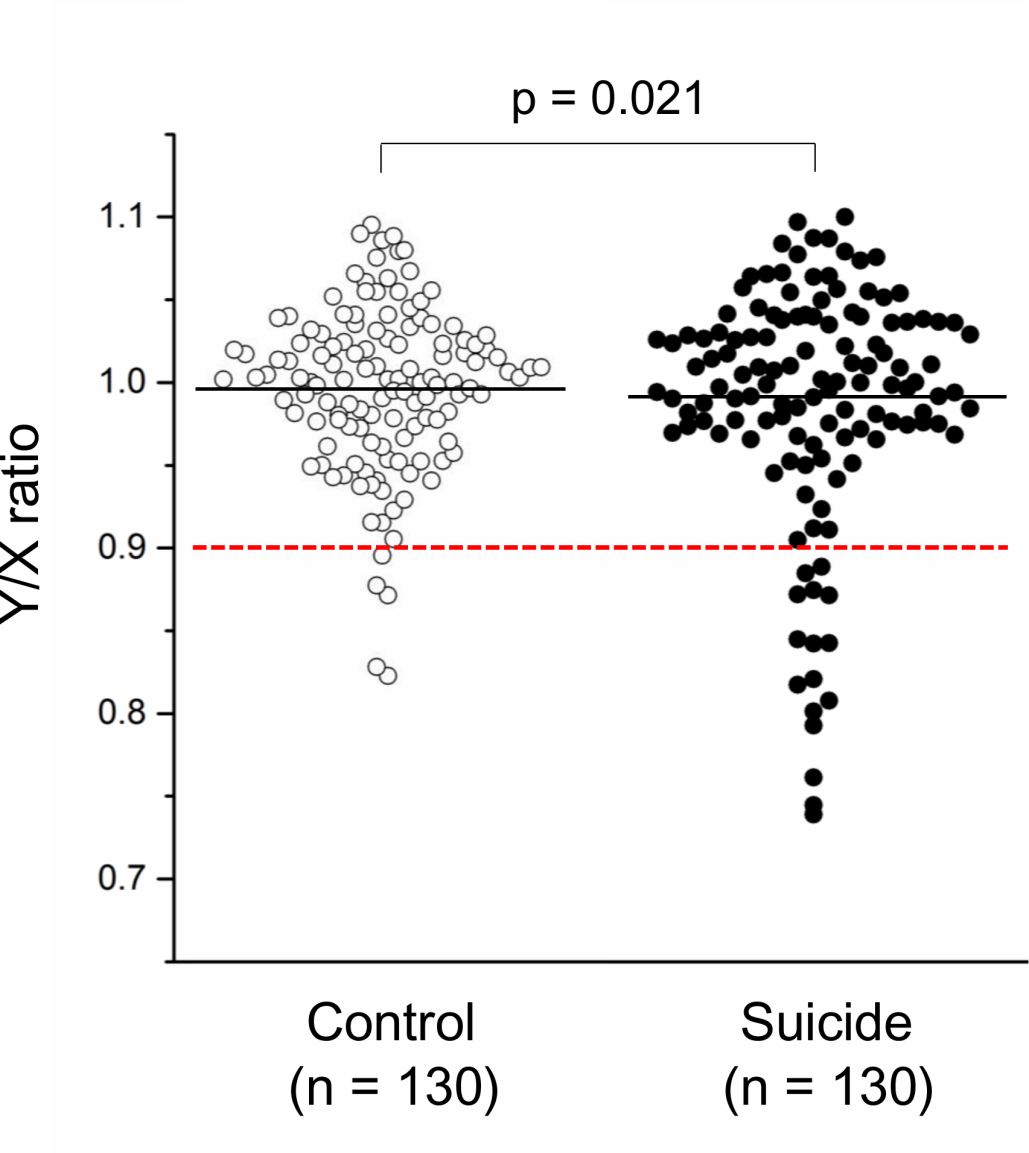
Dot plot of LOY in blood of suicide completers and healthy controls. The black line represents the mean of the Y/X ratio in each group. The red dashed line shows the threshold for LOY (Y/X ratio < 0.9). The p-value was derived from multiple logistic regression analysis controlling for age.

The relative amount of chromosome Y (Y/X ratio) in blood of suicide completers and controls is shown in Fig 1.

**Fig 2:**
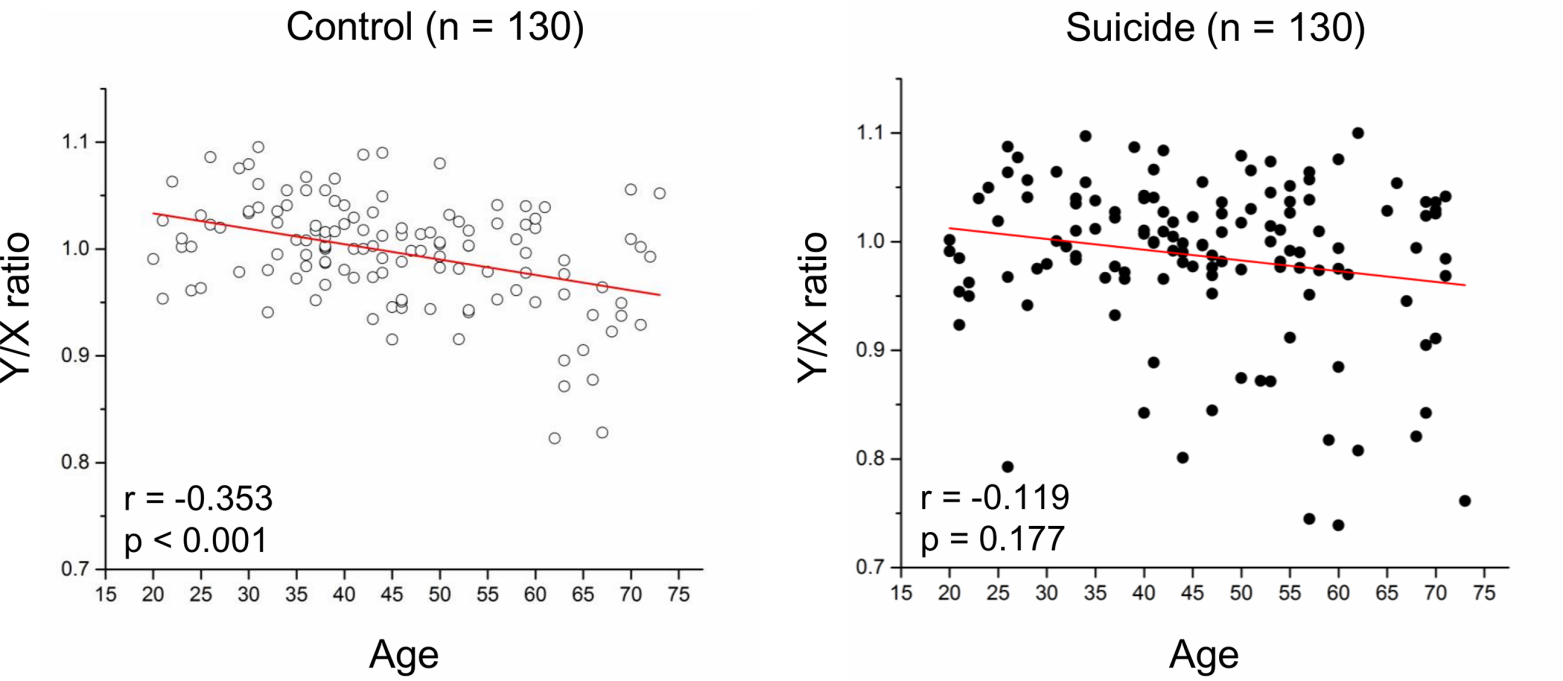
Relationship between age and LOY in blood of suicide completers and healthy controls. All p-values and r values were calculated by the Spearman’s rho test.

Logistic regression analyses showed that LOY in peripheral blood was more frequent in suicide completers than that found in controls (odds ratio [OR] = 3.51, 95% confidence interval [95% CI] = 1.25–9.89, p = 0.018), a finding which remained significant after controlling for age (OR = 3.50, 95% CI = 1.21–10.10, p = 0.021) (Table 2). All 5 control subjects with LOY were over 60 years of age, whereas 10 of the 16 suicide completers with LOY were under 60 years of age (Table 3). The number of current or former smokers in our 16 suicide cases with LOY was only 4, a proportion which is either comparable to or lower than that found in all suicide completes in this study and the general Japanese population [23]. Spearman correlation coefficients showed that normal age-dependent LOY was seen in healthy controls (r = -0.353, p < 0.001), but not in suicide completers (r = -0.119, p = 0.177) (Fig 2).

**Table 2:** Binary and multiple logistic regression analyses of LOY in peripheral blood of suicide completers and controls.

All samples (n = 260)	LOY (Y/X ratio < 0.9)							
	B^a^	p^a^	OR^a^	95% CI^a^	B^b^	p^b^	OR^b^	95% CI^b^
Phenotype (Suicide vs. Control)	1.26	**0.018**	3.51	1.25–9.89	1.25	**0.021**	3.50	1.21–10.10
Age (years)	0.07	**<0.001**	1.07	1.03–1.11	0.07	**<0.001**	1.07	1.03–1.11

Abbreviations: LOY, loss of chromosome Y; OR, odds ratio; CI, confidence interval.

^a^Statistical values were derived from binary logistic regression analyses. B represents the unstandardized partial regression coefficient. p-values shown in bold are significant at < 0.05.

^b^Statistical values were derived from multiple logistic regression analysis adjusted for phenotype (Suicide vs. Control) and age. B represents the unstandardized partial regression coefficient. p-values shown in bold are significant at < 0.05.

**Table 3:** Demographics of suicide completers and healthy controls with LOY in blood.

Suicide completers with LOY (n = 16)			Healthy controls with LOY (n = 5)	
Age (yo)	Y/X ratio	Smoking^a^	Age (yo)	Y/X ratio
26	0.793	Never	62	0.823
40	0.842	Never	63	0.871
41	0.889	Current	63	0.896
44	0.801	Never	66	0.877
47	0.845	Never	67	0.828
50	0.874	Never		
52	0.871	Current		
53	0.871	Never		
57	0.745	Never		
59	0.817	Former		
60	0.739	Never		
60	0.885	Never		
62	0.808	Never		
68	0.821	Current		
69	0.842	Never		
73	0.761	Never		

Abbreviations: LOY, loss of chromosome Y; yo, years old. LOY determined as a chromosome Y/chromosome X ratio < 0.9.

^a^ For smoking status, “Never” refers to an individual who has never been a cigarette or cigar smoker, while “Former” indicates an individual who had smoked in his or her lifetime but who was not a smoker at the time of suicide, and “Current” refers to an individual who was a smoker at the time of suicide.

We also investigated whether PMI affect detection of LOY using binary logistic regression analysis, and found no association between PMI and LOY in post-mortem samples of suicide completers (OR = 1.28, 95% CI = 0.72–2.27, p = 0.399). SNP analyses revealed a nominal significant difference in the genotype distribution and the allele frequency of rs12448368 (p = 0.028 and p = 0.019, respectively), although no SNPs investigated was significantly associated with LOY after correction for multiple comparisons (S2 Table). Significant LOY in the blood of suicide completers remained after controlling for not only age but also the effects of the five SNPs (OR = 3.05, 95% CI = 1.04–8.99, p = 0.043) (S3 Table). Further, we investigated whether smoking status affects LOY in suicide completers, using logistic regression analysis with age, and found no association between smoking status and LOY in blood samples of the suicide completers (current smokers vs. never/former smokers, OR = 1.01, 95% CI = 0.25–4.09, p = 0.993; current/former smokers vs. never smokers, OR = 1.25, 95% CI = 0.34–4.56, p = 0.737; current smokers vs. former smokers vs. never smokers, OR = 1.07, 95% CI = 0.54–2.12, p = 0.856).

### LOY analysis in brain tissue

Multiple linear regression analysis found age-dependent LOY in DLPFC brain samples similar to that found in blood (B = -0.002, p = 0.015), although neither completed suicide nor PMI had evidence of an association with LOY (Table 4).

**Table 4:** Multiple regression analysis of LOY in post-mortem brain of suicide completers and controls.

All samples (n = 26)	Y/X ratio		
	B^a^	s.e.	p^b^
Phenotype (Suicide vs. Control)	-0.004	0.021	0.856
Age (years)	-0.002	<0.001	**0.015**
PMI (hours)	<0.001	0.001	0.523

Abbreviations: s.e., standard error; PMI, post-mortem interval.

^a^B represents the unstandardized partial regression coefficient.

^b^p-value shown in bold is significant at < 0.05.

## Discussion

In this study, we found from our statistical analysis that a significant number of middle-aged male suicides in our Japanese cohort had LOY beyond natural age effect. To our knowledge, our study is the first to demonstrate that LOY in peripheral blood is associated with suicide completion. To date, similar to our study, several studies have proposed that aberrant LOY in peripheral blood may be involved in the pathophysiology of diseases in tissues other than peripheral blood [14,17]. In this study, we conducted additional post-mortem brain analyses to directly investigate the relationship between brain LOY and completed suicides in which the main diathesis is in specific brain regions. Unfortunately, we did not find evidence of an association between LOY in the DLPFC of brain and suicides in our post-mortem cohort. However, the number of brain samples in our study were few (n = 26) compared to that of our blood samples (n = 260) and our analysis did not include other brain regions implicated in the pathophysiology of suicide. Therefore, we could not exclude the possibility of the association between LOY and the brain pathophysiology of completed suicide, and we posit that our findings may contribute towards an improved understanding of the mechanisms underlying sex-related differences in suicides associated with sex chromosome abnormalities. In particular, sex determining region Y (*SRY*) on the Y chromosome regulates dopamine biochemistry and function in the male brain [6], and also regulates expression of monoamine oxidase A (*MAOA*) [24], which is located on the X chromosome and has a role in degrading amine neurotransmitters such as dopamine. There is increasing evidence that the dopaminergic system and MAOA have important roles in suicide-related pathophysiology in at least a subgroup of affected individuals [1,25]. In addition, several studies reported that polymorphisms in dopamine-related genes [e.g., dopamine active transporter 1 (*DAT1*), dopamine receptor D2 (*DRD2*) and D4 (*DRD4*)] and *MAOA* are associated with violent behaviors (particularly in male cohorts) [26–30]. According to Dumais et al. [31], all suicide completers in our peripheral blood cohort used violent methods, which is consistent with epidemiological evidence that men more often use high-violence methods (such as hanging and jumping) compared to women. Because this higher lethality in male suicides may be associated with aberrant changes of dopaminergic genes and *MAOA*, we propose that expression levels of dopamine-related genes and *MAOA* in individuals with significant LOY should be further investigated. In addition to our investigation whether LOY is associated with suicide completion, we also investigated whether there was evidence of an association between age and LOY in our cohorts. We found that our control subjects showed an age-dependent LOY similar to that found in previous reports [12,20], whereas we found no such age-related LOY in suicide completers from our cohort. This age-related difference between groups may be because of the inclusion of suicide completers in our cohort having aberrant LOY beyond normal age-dependent loss.

Further, to investigate whether LOY occurs in brain, the relative amount of Y chromosome was determined using post-mortem brain tissue samples of the DLPFC from suicidal and control brains. DLPFC has been shown to be one of the brain region implicated in suicidal vulnerability. Structural and functional neuroimaging studies of patients with suicidal behaviors demonstrated aberrant volume reductions and dysfunction in the frontal cortex, primarily in the DLPFC as well as the orbitofrontal cortex and ventrolateral PFC [32]. The DLPFC is deeply involved in cognitive control and emotional regulation, both of which are involved in suicide-related pathophysiology [33]. To date, no prior study investigated LOY in brain regions, even with cohorts without psychiatric problems. Although we found no evidence of an association between LOY in the DLPFC of brain and completed suicide, we provided the first demonstration that age-dependent LOY occurs not only in blood cells but also in brain. Based on our findings, we propose that various factors, such as aging, smoking, physical diseases, or psychological problems, alter the frequency of mosaic LOY in some brain regions as well as in blood cells. Future studies using post-mortem brains of suicide completers are warranted to determine whether LOY in other brain regions, such as the orbitofrontal cortex, ventral PFC, hippocampus, or amygdala, is correlated with suicidal vulnerability.

We could not exclude that our results were caused by low power, stratification in the samples, or some other complexity in our data, then we identified several limitations in the present study. The first regards the use of post-mortem samples. Although only subjects with certain clinical information prior to death were selected for inclusion, we are unable to exclude possible confounding effects of PMI and biological degradation of post-mortem samples in our results. However, because we found no evidence of an association between PMI and LOY in post-mortem blood and brain samples in this study, we posit that our results were not significantly affected by the condition of the post-mortem samples. A second limitation of our study is that clinical information regarding smoking status, a factor known to affect LOY [13], was not available for our control individuals. However, we found no evidence of bias in the smoking status of suicide completers with LOY in blood compared to those without LOY in our cohort, as well as in the general Japanese male population (Table 3). In addition, we showed that smoking status had no statistically significant effect on the detection of LOY in blood samples of suicide completers. These findings collectively indicate that our results were not significantly influenced by smoking status of the subjects. However, further investigation with detailed information regarding smoking status (i.e. cigarettes per day, duration of smoking) of controls are warranted. Third, the sample sizes for postmortem blood and brain LOY assessments were too small to detect true evidence for the disappearance of normal age-dependent LOY in the suicidal blood and age-dependent LOY in the DLPFC. Fourth, our results of the SNP analyses led us to expect that some of the LOY-associated SNPs previously identified by European GWASs would have the similar effects on LOY in the Japanese population. However, there are other previously reported LOY-associated polymorphisms that we did not investigate in the current study and thus, further comparisons with larger sample sizes are needed to better identify and characterize all culprit loci [15,16]. In addition, future studies should focus on not only suicide completes but also “suicide attempters,” who are more common, to more robustly predict completed suicide [34].

In conclusion, this is the first study to not only investigate and provide evidence demonstrating aberrant chromosome Y loss in adult male suicide completers, but we also discovered that age-dependent LOY occurs in blood and in certain brain regions. Our findings shed light on further research into LOY towards an improved understanding between suicide-related pathophysiology and brain-specific chromosomal abnormalities.

## Supporting information

S1 Table Primer sequences and polymerase chain reaction conditions. (PDF)

S2 Table Genotype and allele frequencies of the LOY-associated SNPs in our blood samples. (PDF)

S3 Table Multiple logistic regression analysis of LOY in peripheral blood of suicide completers and controls with the LOY-associated SNPs as covariates. (PDF)
