# Supplementary material for: Loss of chromosome Y in blood, but not in brain, of suicide completers
Source: PLoS One. 2018 Jan 4;13(1):e0190667. doi: 10.1371/journal.pone.0190667 (PMC5754120; doi:10.1371/journal.pone.0190667)
Supplement: S1 Table — (PDF) [file pone.0190667.s001.pdf]

S1 Table. Primer sequences and polymerase chain reaction conditions.

Locus	Primers (5'-3')
AMELY<br>*AMELX*	F: CCCTGGGCTCTGTAAAGAATAGTG<br>R: ATCAGAGCTTAAACTGGGAAGCTG<br>95 °C for 5 min followed by 28 cycles of 95 °C for 1 min, 58 °C for 1 min, and 72 °C for 1.5 min
TAF9B<br>Homologous sequence	F: TTTGACAGGTAGTTTTGGGTCA<br>R: TGGTTTTGCCTAGGTCCAGT<br>95 °C for 5 min followed by 28 cycles of 95 °C for 1 min, 58 °C for 1 min, and 72 °C for 1.5 min
MYPT2<br>Homologous sequence	F: CTCACTACATGACATTCAGG<br>R: GTTTCTTCCCAGTATCTAGTACAGTGC<br>95 °C for 5 min followed by 28 cycles of 95 °C for 1 min, 58 °C for 1 min, and 72 °C for 1.5 min

Abbreviations: *AMELY*, amelogenin, Y-linked; *AMELX*, amelogenin, X-linked; *TAF9B*, TATA-box binding protein associated factor 9b; *MYPT2*, myosin phosphatase target subunit 2; min, minutes.
