# Supplementary material for: Loss of chromosome Y in blood, but not in brain, of suicide completers
Source: PLoS One. 2018 Jan 4;13(1):e0190667. doi: 10.1371/journal.pone.0190667 (PMC5754120; doi:10.1371/journal.pone.0190667)
Supplement: S2 Table — (PDF) [file pone.0190667.s002.pdf]

S2 Table. Genotype and allele frequencies of the LOY-associated SNPs in our blood samples.

SNP ID[a] / Position[a]	Phen[b]	Genotype distribution			Minor allele		p-value[c]			Power[d]	OR (95% CI)	MAF in JPT[a]	Nearest gene[a]	GWAS p-value in previous studies[e]
		MM	Mm	mm	MAF	Allele	HWE	Genotype	Allelle					
rs13191948	Case	15	6	0	0.143	T	1.000	0.288	0.261	0.172	1.69	0.077	*CCDC162P*	2.20×10^{-19}
Chr6: 109634599	Con	201	33	5	0.090		0.057		(0.797)		(0.67-4.23)			
rs4721217	Case	10	9	2	0.310	T	1.000	0.105	0.098	0.198	0.57	0.404	*MAD1L1*	3.50×10^{-14}
Chr7: 1973579	Con	77	113	49	0.441		0.590		(0.398)		(0.29-1.12)			
rs2887399	Case	18	3	0	0.071	T	1.000	0.912	0.912	0.056	1.07	0.029	*TCL1A*	1.37×10^{-10}
Chr14: 96180695	Con	207	32	0	0.067		0.658		(1.000)		(0.31-3.66)			
rs12448368	Case	11	7	3	0.310	C	0.525	0.028	0.019	0.405	2.26	0.212	*CENPN*	7.10×10^{-11}
Chr16: 81044947	Con	171	57	11	0.165		0.067		(0.078)		(1.12-4.55)			
rs11082396	Case	14	6	1	0.190	C	1.000	0.097	0.124	0.190	0.54	0.250	*SETBP1*	1.20×10^{-10}
Chr18: 42080720	Con	108	117	14	0.303		0.021		(0.508)		(0.24-1.20)			

Abbreviations: SNP ID, single nucleotide polymorphism identification; Phen, phenotype; M, major allele; m, minor allele; MAF, minor allele frequency; HWE, Hardy-Weinberg equilibrium; OR, odds ratio; CI, confidence interval; GWAS, genome-wide association study; Con, control; CCDC162P, coiled-coil domain containing 162, Pseudogene; MAD1L1, mitotic spindle assembly checkpoint protein MAD1; TCL1A, T-cell leukemia/lymphoma 1A; CENPN, centromere protein N; SETBP1, SET binding protein 1.

[a] SNP ID number, positions, MAF in Japanese (JPT) populations and nearest gene are available at The 1000 Genomes Project (phase 3) (http://phase3browser.1000genomes.org/index.html).

[b] Case means our subjects with loss of chromosome Y (LOY) (n = 21). Con means our subjects without LOY (n = 239).

[c] Genotypic p values were tested with Cochran-Armitage test for trend. Allelic p values were tested with Chi square; corrections for multiple comparisons are in parentheses (for 10,000 permutations).

[d] The power analysis was performed with the PS v2.1.3.1 program.

[e] p-value of rs2887399 was from the GWAS by Zhou et al. [15] and p-values of other SNPs were from the GWAS by Wright et al. [16].
