# Supplementary material for: Loss of chromosome Y in blood, but not in brain, of suicide completers
Source: PLoS One. 2018 Jan 4;13(1):e0190667. doi: 10.1371/journal.pone.0190667 (PMC5754120; doi:10.1371/journal.pone.0190667)
Supplement: S3 Table — (PDF) [file pone.0190667.s003.pdf]

S3 Table. Multiple logistic regression analysis of LOY in peripheral blood of suicide completers and controls with the LOY-associated SNPs as covariates.

All samples (n =260)	LOY (Y/X ratio < 0.9)			
	B[a]	p[a]	OR[a]	95% CI[a]
Phenotype (Suicide vs. Control)	1.12	**0.043**	3.05	1.04-8.99
Age (years)	0.06	**0.001**	1.06	1.02-1.10
rs13191948 (MM/Mm/mm)	0.48	0.331	1.61	0.62-4.24
rs4721217 (MM/Mm/mm)	-0.57	0.135	0.57	0.27-1.19
rs2887399 (MM/Mm/mm)	-0.10	0.890	0.91	0.23-3.56
rs12448368 (MM/Mm/mm)	0.53	0.143	1.69	0.84-3.42
rs11082396 (MM/Mm/mm)	-0.70	0.153	0.50	0.19-1.30

Abbreviations: LOY, loss of chromosome Y; OR, odds ratio; CI, confidence interval; M, major allele; m, minor allele.

[a]Statistical values were derived from multiple logistic regression analysis adjusted for phenotype (Suicide vs. Control), age, and the genotype of the five SNPs. B represents the unstandardized partial regression coefficient. p-values shown in bold are significant at < 0.05.
